# Stereotactic body radiotherapy with volumetric intensity-modulated arc therapy and flattening filter-free beams: dosimetric considerations

**DOI:** 10.1007/s00066-023-02181-8

**Published:** 2023-12-13

**Authors:** Mark Christoph Brekner, Detlef Imhoff, Claus Rödel, Natalie Filmann, Jörg Licher, Ulla Ramm, Janett Köhn

**Affiliations:** 1 Department of Radiation Oncology, University Hospital, Johann Wolfgang Goethe University Frankfurt, Theodor Stern Kai 7, 60590 Frankfurt, Germany; 2 Institute for Biostatistics and Mathematical Modeling, University Hospital, Goethe University, 60590 Frankfurt, Germany

**Keywords:** SBRT, FFF, VMAT, MU, Energy-matched

## Abstract

**Purpose:**

The present study comparatively evaluates the impact of energy-matched flattening filter-free (FFF) photon beams with different energy levels on the physical-dosimetric quality of lung and liver stereotactic body radiotherapy (SBRT) treatment plans.

**Methods:**

For this purpose, 54 different lung and liver lesions from 44 patients who had already received SBRT combined with volumetric modulated arc therapy (VMAT) were included in this retrospective planning study. Planning computed tomography scans already available were used for the renewed planning with 6 MV, 6 MV-FFF, 10 MV, and 10 MV-FFF under constant planning objectives. The treatment delivery data, dosimetric distributions, and dose–volume histograms as well as parameters such as the conformity index and gradient indices were the basis for the evaluation and comparison of treatment plans.

**Results:**

A significant reduction of beam-on time (BOT) was achieved due to the high dose rates of FFF beams. In addition, we showed that for FFF beams compared to flattened beams of the same energy level, smaller planning target volumes (PTV) require fewer monitor units (MU) than larger PTVs. An equal to slightly superior target volume coverage and sparing of healthy tissue as well as organs at risk in both lung and liver lesions were found. Significant differences were seen mainly in the medium to lower dose range.

**Conclusion:**

We found that FFF beams together with VMAT represent an excellent combination for SBRT of lung or liver lesions with shortest BOT for 10 MV-FFF but significant dose savings for 6 MV-FFF in lung lesions.

**Supplementary Information:**

Additional tables and a list of abbreviations are provided in the supplementary data (10.1007/s00066-023-02181-8).

## Introduction

Stereotactic body radiotherapy (SBRT) plays an important role in modern-day oncology. Starting out in research settings only, SBRT has evolved over time with technical advancements and growing clinical knowledge in the fields of image-guided radiotherapy, motion management, and medical linear accelerators (linacs). Thus, during the past two decades, SBRT has become a commonly used procedure for the treatment of various tumor entities [1].

Especially in the therapy of early-stage non-small cell lung cancer (NSCLC), several clinical studies have delivered promising results on SBRT showing high local control rates with acceptable toxicity and good feasibility [2, 3]. Today, SBRT is no longer merely a guideline-recommended therapeutic option for medically inoperable patients [4, 5], but it also shows encouraging results in the treatment of operable patients with early-stage NSCLC [6, 7]. Furthermore, numerous clinical studies showed that SBRT is a good noninvasive treatment option for hepatic tumors as well as in patients with oligometastatic disease of the lung and/or liver [8–11].

A highly conformal dose distribution with a steep dose fall-off around the planning target volume (PTV) and a highly precise dose delivery are essential for SBRT. In addition to patient positioning, “in-room” imaging and respiratory motion management (ABC = active breathing coordinator, breath-gating), beam-on time (BOT), and treatment time are important factors for precise delivery of SBRT in pulmonary and hepatic tumor sites. Since SBRT is performed in one or few fractions [12], a high single dose per fraction must be applied to the PTV. Consequently, the BOT and treatment time are extended while increasing the probability of intrafractional movements of the patient and of the tumor itself [13]. This is important, especially for internal organ and tumor movement related to respiration or peristalsis, as in the case of lung or liver lesions [14]. Shortening the BOT not only minimizes intrafractional movements, but also improves patient comfort and the feasibility of respiratory motion management techniques for patients who are often pre-diagnosed with cardiopulmonary diseases [15].

The implementation of volumetric modulated arc therapy (VMAT) has already enabled a more efficient delivery of highly conformal dose distribution compared to conventional intensity-modulated radiotherapy (IMRT) for SBRT [16]. By operating a linac flattening filter-free (FFF), the dose rate can be increased in the center of the beam by up to approximately four times, which could further shorten the BOT by more than 50% [17]. Other favorable dosimetric characteristics of the FFF beams include the reduction of head scatter, head leakage, leaf transmission, and electron contamination resulting in decreased peripheral doses [18–20]. Additionally, a diminutive off-axis spectral dependence is observed for FFF beams [21]. Due to the promising advantages of this treatment technique, especially in combination with VMAT, many clinical sites have introduced FFF linacs.

As noted by Hansen et al. [22], a variety of vendor- and patient-related factors play a critical role in treatment delivery as well as plan quality. In previous studies only a small number of patients with specific tumor entities and mostly bigger target volumes were investigated comparing a few selected irradiation techniques. Moreover, the majority of published treatment planning studies on dosimetric evaluation of FFF beams used for SBRT of pulmonary or hepatic lesions have been performed with linacs in which the beam energy was not energy-matched [23–31]. In the case of energy-matched FFF beams, the beam quality is matched with the flattened beams so that beam quality indices such as the D10 are the same for FFF and unflattened beams. For this purpose, the electron energy of the FFF beams is increased accordingly, which effectively results in an energy difference between matched FFF beams and not-matched FFF beams [32].

We conducted a retrospective treatment planning study of SBRT of lung and liver lesions in different tumor entities with a wide range of target volume sizes, especially patients with lung lesions, comparing 6‑MV and 10-MV beams with and without flattening filter (FF) under VMAT conditions. The purpose was the comparison of the physical-dosimetric quality of the generated treatment plans. In particular, we evaluated whether a reduction in the applied radiation dose to the surrounding organs at risk (OAR) and/or a reduction of the irradiation time can be achieved with a constant exposure of the OAR at dosimetric coverage of the target volume by varying the radiation technique and the photon energy.

## Materials and methods

### Patient and tumor characteristics

Since 2012, SBRT of malignant pulmonary and hepatic lesions has been performed at the Department of Radiotherapy and Oncology at our university hospital. Institutional review board approval was obtained for this single-center, retrospective study including all adult patients treated with VMAT-SBRT from 2012 until the end of 2018.

A total of 44 patients with 54 malignant pulmonary (*n* = 37) and/or hepatic (*n* = 17) lesions were evaluated. The patient and tumor characteristics are summarized in Table 1. This study included a heterogeneous patient population with a variety of tumor localizations, histologies, and volumes, which were consequently treated with individualized dose and fractionation schemes.

**Table 1:** Patient and tumor characteristics

**Patients (*n*** **=** **44)**			
*Male* 1 pulmonary lesion 2 pulmonary lesions 3 pulmonary lesions 1 hepatic lesion 2 hepatic lesions 1 pulmonary and 1 hepatic lesion 2 pulmonary and 1 hepatic lesions	*n* *=* *29 (65.9%)* *n* = 16 *n* = 1 *n* = 2 *n* = 7 *n* = 1 *n* = 1 *n* = 1	*Female* 1 pulmonary lesion 2 pulmonary lesions 1 hepatic lesion	*n* *=* *15 (34.1%)* *n* = 8 *n* = 1 *n* = 6
**Treatment plans (*n*** **=** **54)**			
**Malignant pulmonary lesions (*n*** **=** **37) (68.5%)**		**Malignant hepatic lesions (*n*** **=** **17) (31.5%)**	
Median age	73 years (52–86 years)	Median age	63 years (37–84 years)
Peripheral location Central/superior location Right pulmonary lobe Left pulmonary lobe	*n* = 21 *n* = 16 *n* = 21 *n* = 16	Left hepatic lobe (segment 1–4) Right hepatic lobe (segment 5–8)	*n* = 8 *n* = 9
NSCLC SCLC Pulmonary metastases Not clearly assignable	*n* = 8 *n* = 1 *n* = 23 *n* = 5	HCC Hepatic metastases Not clearly assignable	*n* = 5 *n* = 10 *n* = 2
GTV: median = 3.9 ccm (0.4–40.4 ccm) PTV: median = 14.4 ccm (4.0–94.1 ccm)		GTV: median = 12.6 ccm (0.6–142.4 ccm) PTV: median = 46.0 ccm (15.7–223.9 ccm)	

*NSCLC* non-small cell lung carcinoma, *SCLC* small cell lung carcinoma, *HCC* hepatocellular carcinoma, *PTV* planning target volume, *GTV* gross tumor volume

### Planning and treatment characteristics

Patients were scanned according to the department computed tomography (CT) protocol. Usually, vacuum bags were used for immobilization, and abdominal compression was required in 19 cases. Almost all patients were scanned and treated using an ABC system. If patients were not suitable for ABC, which was the case for 19 patients, a 4D-CT scan was used. For lung cases, the PTV was generated from the gross tumor volume (GTV) with a margin of 5 mm. For patients with 4D-CT scan the internal target volume (ITV) was delineated from the maximal intensity projection out of ten breathing phases. The PTV included ITV with an expansion of 5 mm. the PTV size ranged from 2.6 ccm to 94.1 ccm with a median of 14.4 ccm. For hepatic cases, the GTV was delineated from magnetic resonance images of the macroscopic tumor. The clinical target volume (CTV) was generated from the GTV with a 5-mm margin and the PTV from the CTV with a 3-mm margin. If the PTV was generated from the ITV, the margin was 5–7 mm. The PTV size ranged from 15.7 ccm to 223.9 ccm with a median of 46 ccm.

Treatment plans were generated with the treatment planning system (TPS) Oncentra Masterplan (Elekta, Stockholm, Sweden, v4.5.3) utilizing Raysearch optimizer and a collapsed cone algorithm. The optimizer works as a sequential quadratic programming algorithm solving general non-linear optimization problems. Penalty functions were used for variable bounds. The optimizer went through 50 iterations. Constraints are not possible in VMAT optimization.

Objectives were selected according to the main goal of optimization to maximize the coverage of GTV and to cover 95% of PTV with the prescribed surrounding isodose. The percentage of the surrounding isodose was defined by the value of maximum dose. Organ-specific dose objectives were defined only in the case that the OAR was located near the PTV and a limitation was necessary. A surrounding dose fall-off function was used in almost all cases to limit the dose to OAR. We applied VMAT to patients with ABC or low tumor movement, using two or three coplanar arcs with individual lengths. If necessary, a sector was avoided to spare OAR. Objectives for all four energies were kept the same.

The dose calculation grid size was 2 mm. The maximum dose rate was allowed for each beam (MU = monitor unit; 6 MV with 600 MU/min, 10MV with 400 MU/min, 6 MV-FFF with 1800 MU/min, 10 MV-FFF with 2100 MU/min). The maximum field size for the multi-leaf collimator (MLC) is 40 × 40 cm^2^ with a leaf width of 5 mm at the isocenter plane. The 6‑MV and 10-MV beams with and without FF were energy-matched and turned to hold the same relative dose at a depth of 10 cm.

The prescription dose was individual for every patient depending on the primary tumor, the histology, as well as the tumor size and location. For patients with lung lesions, the most frequently used schemes were 3 × 15 Gy and 3 × 18 Gy to 70–83% isodose (*n* = 21). For seven patients, schemes of 5 × 10–11 Gy to 75–80% isodose were applied and for four patients 8 × 6–7 Gy to 65–70% isodose. For other patients, individual schemes were used. The final dose to the PTV ranged between 33 and 56 Gy to the 65–83% isodose with three to ten fractions. For patients with liver lesions, the mainly used scheme was 3 × 15 Gy to 75–83% isodose (*n* = 7). Individual schemes with three to ten fractions were used in other patients. The final dose to the PTV ranged between 30 and 54 Gy to the 63–100% isodose with three to ten fractions.

### Data collection and plan quality characteristics

Based on the existing planning CT images and the clinical treatment plans, four new treatment plans including 6 MV, 6 MV-FFF, 10 MV, and 10 MV-FFF with VMAT were generated for the Elekta VersaHD. To avoid performance bias, the same optimization objectives were applied to all generated treatment plans. The geometrics of the beams also remained unchanged. Objectives for target volumes were modified only when PTV coverage changed dramatically. For standardization purposes, the already existing planning CT images were again overlaid consistent with all relevant OAR and other auxiliary contours for the dosimetric evaluation of the irradiation plans. All treatment plans generated were clinically acceptable for SBRT and achieved the prescribed dose and tumor coverage, while adhering to the specified dose constraints in most cases, regardless of the treatment technique.

The treatment delivery data, dosimetric distributions, and dose–volume histograms as well as parameters describing the plan quality such as the conformity index (CI) and gradient indices (GI) served as the basis for the evaluation and comparison of the treatment plans.

For all treatment plans the total number of MUs and the BOT were collected from the TPS for describing the treatment delivery. These data were used to calculate the delivery dose rates (MU/min). To compare the target volume coverage, doses to the GTV and PTV as well as the CI according to formula (1) [33] were collected for all plans. In addition to the captured GIs according to formula (2) [34] and (3) [35], 2‑mm-thick shells were set up in 1‑cm, 2‑cm, and 3‑cm distance to the PTV surface to determine the dose fall-off around the PTV. The collected dose values in these shells were evaluated in relation to the prescribed dose.

1 $$\mathrm{CI}=\frac{PTV\times PIV}{{TV_{\mathrm{PIV}}}^{2}}\left(\text{reciprocal Paddick CI [33]}\right)$$

2 $$\mathrm{GI}_{P{\&}L}=\frac{\mathrm{PIV}_{50{\%}\mathrm{PIV}}}{PIV}\left(\text{Paddick and Lippitz [34]}\right)$$

3 $$\mathrm{GI}_{\text{RTOG}}=\frac{\mathrm{PIV}_{50{\%}\mathrm{PIV}}}{PTV}\left(\text{RTOG 0915 [35]}\right)$$

PTV: = planning target volume
PIV: = prescription isodose volume
TV_PIV_: = target volume within the PIV
PIV_50%PIV_: = volume of half the prescription isodose

To describe the dose exposure of healthy tissue, integral dose parameters were obtained. The auxiliary contour for the integral dose was defined as the volume up to the outer contour of the patient’s body and extending to 4 cm above and below the PTV, excluding the PTV itself.

**Fig. 1:**
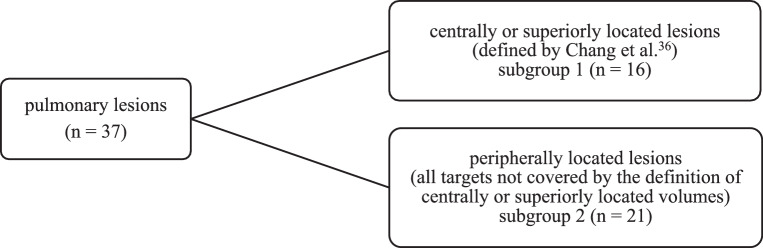
Subgroup division. Division of pulmonary lesions into subgroups based on their localization

The pulmonary target volumes were divided into two subgroups as defined by Chang et al. [36] according to their localization and the resulting proximity to the OAR (Fig. 1). The hepatic lesions were not further subdivided since no uniform groups could be formed due to previous surgery or variations of the adjacent OAR.

All serial OAR were delineated to 3 cm above and below the PTV if they were imaged in this range of the CT. Parallel OAR as well as the heart were completely delineated if they were fully mapped in the planning CT, otherwise they were not evaluated. Two patients with lung lesions had already undergone lobectomy; consequently, dose values of the contralateral lung could not be considered.

Further information on which dosimetric parameters were evaluated in the pulmonary treatment plans, the two subgroups, and the hepatic plans can be found in the supplementary data.

### Statistical analysis

Statistical analyses were performed with R (version 4.0.2, R Core Team, 2020, Vienna, Austria, packages nparLD, MASS, coin, pROC, and multcomp). Continuous variables are presented as medians with quartiles. The WilcoxonMann–Whitney *U *test was used for comparisons of quantitative and ordinal variables at baseline or at low threshold. For comparisons of the treatment plans, the Friedman test was used whereby post hoc comparisons were evaluated according to the exact Wilcoxon sign rank test. Post hoc *p *values were adjusted according to Bonferroni. Spearman correlation was used to analyze the correlation between MUs and PTV. Thresholds for PTVs in the consideration of MU ratios were estimated via receiver operating characteristics analysis. All tests were two-sided and values of *p* < 0.05 were considered statistically significant.

## Results

The statistical summary of the absolute dosimetric parameters and treatment delivery data evaluated for each technique as well as the comprehensive results of the pairwise comparisons are available in the supplementary data.

Each result reported below is statistically significant (*p* < 0.05) unless otherwise described.

**Fig. 2:**
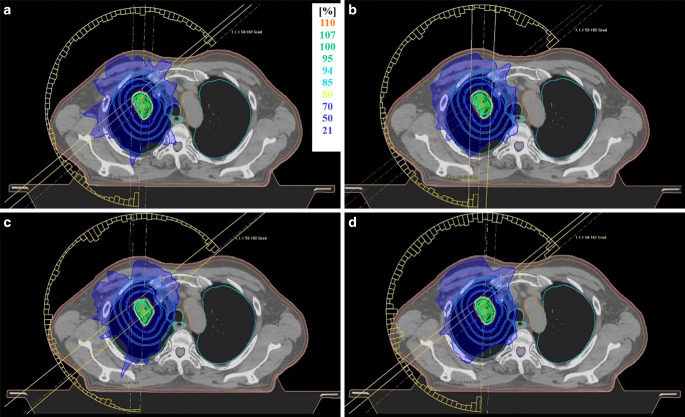
Comparison of dose distributions between the four techniques studied. Exemplary representation of the dose distribution of the four treatment techniques investigated for a pulmonary lesion: **a** 6 MV, **b** 6 MV-FFF, **c** 10 MV, **d** 10 MV-FFF (*FFF* flattening filter free)

An exemplary representation of the dose distribution of the four techniques investigated for a patient with lung disease is shown in Fig. 2.

### Pulmonary lesions

**Fig. 3:**
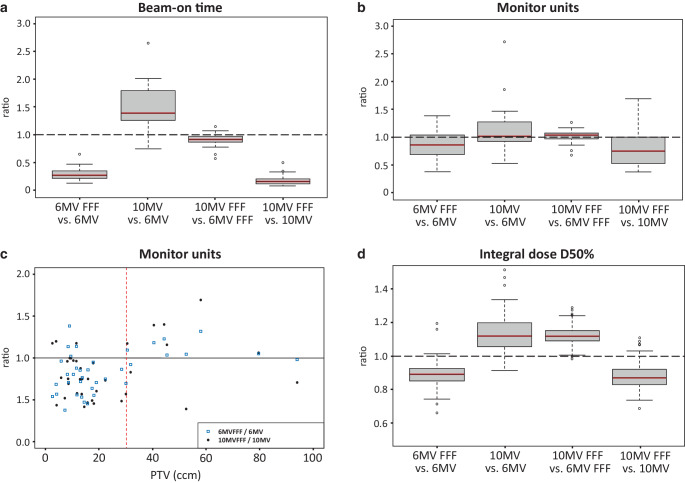
Treatment delivery data of the pulmonary lesions. **a**, **b**, **d** Pairwise relative comparison for all four treatment techniques: **a** beam-on time, **b** monitor units (MU), **d** integral dose D50%. **c** MU ratio for 6 MV/6 MV-FFF and 10 MV/10 MV-FFF as a function of PTV. *Dashed red line*: PTV cut-off value for both comparisons = 30.29 ccm

Treatment delivery data showed that due to the higher dose rates of the FFF beams compared to the flattened beams, a remarkable reduction of the BOT for FFF beams can be achieved (Fig. 3a). Overall, 10 MV-FFF showed the shortest BOT of all four techniques. A mean time saving of 71.7% and 83.7% was demonstrated for 6 MV-FFF and 10 MV-FFF, respectively, compared to the same energy with FF in each case.

The total MUs were mostly lower for FFF beams compared to flattened beams with the same energy level. By contrast, no differences in MUs could be found in the two other comparisons (Fig. 3b).

A significant correlation between the MU ratio and the PTV could be shown for the 6 MV-FFF/6 MV ratio (Spearman’s rho = 0.39, *p* = 0.02), but not for the 10 MV-FFF/10 MV ratio (Spearman’s rho = 0.01, *p* = 0.9). Based on the two calculated MU ratios of 6 MV-FFF/6 MV and 10 MV-FFF/10 MV, we were able to show that for smaller target volumes FFF beams usually require fewer MUs than flattened beams of the same energy level. With increasing PTV on the other hand, FFF beams require more MUs than flattened beams. For both ratios considered, a PTV cut-off value of 30.3 ccm was calculated (Fig. 3c).

The GTV and PTV coverages showed comparable dose distributions, demonstrating a slightly better GTV coverage (D50%, Dmean) for FFF beams and a better PTV coverage (D50%, D95%; *p* = 0.06) for 10 MV-FFF versus 10 MV. Furthermore, 6 MV-FFF showed a somewhat better D50% of PTV compared to 10 MV-FFF. The differences were ≤ 0.9% for GTV coverage and ≤ 1.2% for PTV coverage and therefore probably not of clinical relevance. No differences could be found in the CIs between the four techniques. The median CIs were between 1.29 and 1.33 (supplementary data).

No differences in all pairwise comparisons between the four treatment techniques were found for the GI_RTOG_. The median GIs_RTOG_ were between 5.41 and 5.86. The GI_P&L_ was higher for 10 MV-FFF than for 6 MV-FFF. The median GIs_P&L_ ranged between 5.08 and 5.59. Statistical analysis of the dose fall-off around the PTV based on the generated shells showed a higher dose fall-off of Dmax in the second and third shell for 6 MV-FFF compared to 6 MV. For 10 MV-FFF compared to 10 MV, a tendency (*p* = 0.06) of a higher dose fall-off of Dmax in the second shell as well as a significantly higher dose fall-off of Dmax in the third shell was also demonstrated (supplementary data).

For the integral dose, the data showed lower D50% and Dmean for FFF beams compared to the flattened beams of the same energy level, and higher dose values for D50% and Dmean for 10-MV techniques compared to 6‑MV techniques (Fig. 3d). The absolute dose values are ≤ 3.28 Gy in terms of median values.

**Fig. 4:**
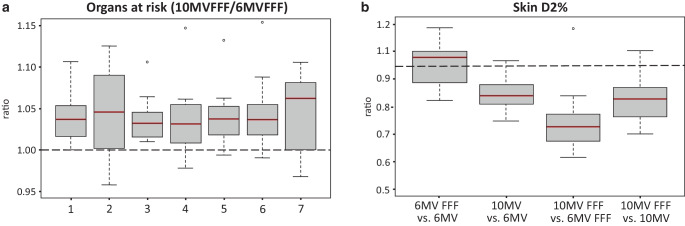
Organs at risk (OAR) of the pulmonary lesions. **a** OAR ratio 10 MV-FFF/6 MV-FFF for central/superior pulmonary lesions: *1* = total lung Dmean, *2* = total lung V5Gy, *3* = tumorous lung Dmean, *4* = tumorous lung V20Gy, *5* = tumorous lung V12.5 Gy, *6* = tumorous lung V10Gy, *7* = contralateral lung Dmean. **b** Skin D2% ratio for all four techniques of peripherally located pulmonary lesions

The OAR data of the centrally or superiorly localized pulmonary lesions indicated an improvement especially in terms of dose exposure to the lung, with 6 MV-FFF showing a dose sparing compared to 10 MV-FFF (Fig. 4a). Furthermore, a lower D2% of the trachea for 6 MV-FFF versus 10 MV-FFF was demonstrated. By contrast, a lower dose exposure for 10 MV-FFF versus 6 MV-FFF regarding the skin (D2%) was found.

Moreover, a dose reduction for the total lung (Dmean), tumor-affected lung (Dmean), and proximal bronchial tree (D2%) for 6 MV-FFF versus 10 MV-FFF was shown for peripherally localized pulmonary lesions. The highest dose exposure to the skin (D2%) of the peripherally localized pulmonary lesions was found for 6 MV-FFF, whereas the lowest dose exposure to the skin was obtained at 10 MV-FFF (Fig. 4b).

Comparison between FFF and flattened beams of the same energy revealed an increased skin dose for 6 MV-FFF versus 6 MV for peripherally localized lung lesions. Otherwise, similar or lower dose exposures to OAR were found for FFF beams versus flattened beams (supplementary data).

Because of small numbers of dose values obtained for the brachial plexus and for the heart, such values were analyzed statistically in a descriptive manner only (supplementary data).

### Hepatic lesions

**Fig. 5:**
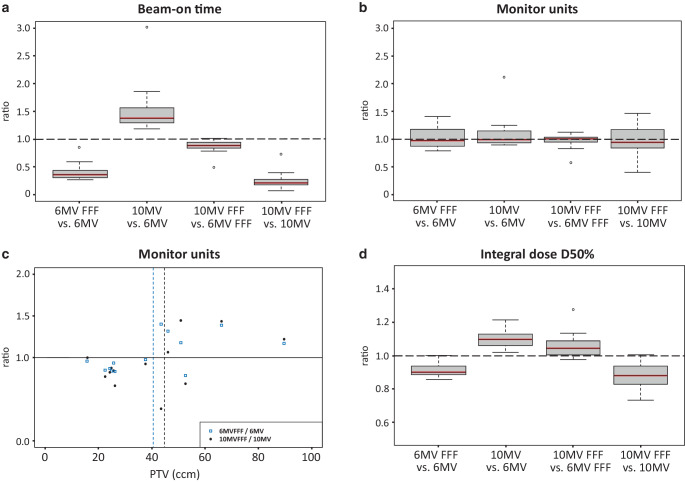
Treatment delivery data of the liver lesions. **a**, **b**, **d** Pairwise relative comparison for all four techniques: **a** beam-on time, **b** monitor units (MU), **d** integral dose D50%. **c** MU ratio for 6 MV/6 MV-FFF and 10 MV/10 MV-FFF as a function of PTV. *Dashed blue line*: PTV cut-off value of 6 MV-FFF/6 MV = 40.57 ccm; *dashed black line*: PTV cut-off value of 10 MV-FFF/10 MV = 44.6 ccm

For hepatic lesions we found comparable results to those for the pulmonary lesions in terms of dose rate and BOT with a remarkable reduction of the BOT for FFF beams. A mean time saving of 63% and 78.4% was demonstrated for 6 MV-FFF and 10 MV-FFF, respectively, relative to the same energy with FF (Fig. 5a).

The MUs showed no differences that depend on the beam selection (Fig. 5b). However, in hepatic lesions, smaller PTVs required also fewer MUs when using FFF beams than larger PTVs did. Liver lesions showed a trend (*p* = 0.08) for 6 MV-FFF/6 MV and a significant (*p* = 0.04) correlation between MU ratios and PTV size for 10 MV-FFF/10 MV. For the MU ratio of 6 MV-FFF/6 MV, a PTV cut-off value of 40.6 ccm was calculated; for the MU ratio of 10 MV-FFF/10 MV a slightly higher cut-off value of 44.6 ccm was found (Fig. 5c).

For liver plans, GTV and PTV coverages also showed somewhat improved GTV coverage for 10 MV-FFF compared to 6 MV-FFF and 10 MV, respectively. Furthermore, related to the PTV, we found a higher D98% for 6 MV-FFF compared to 6 MV, and a trend (*p* = 0.06) toward a higher D98% for 10 MV-FFF relative to 10 MV. No differences between the treatment techniques could be demonstrated for the CI. The median CIs were between 1.16 and 1.19 (supplementary data).

There were no differences in all pairwise comparisons of the two GIs obtained. The median GIs_RTOG_ were between 3.79, and 3.64. The median GIs_P&L_ were between 3.46 and 3.50. For the dose fall-off measured over the generated shells, apart from a higher D50% value for 10 MV versus 6 MV and a comparable tendency for 10 MV-FFF versus 6 MV-FFF, no systematic differences depending on beam selection could be detected (supplementary data).

For the collected dose values of the integral dose, again a lower D50% and Dmean for FFF beams compared to the flattened beams and higher dose values for D50% and Dmean for 10-MV techniques compared to 6‑MV techniques were found (Fig. 5d).

**Fig. 6:**
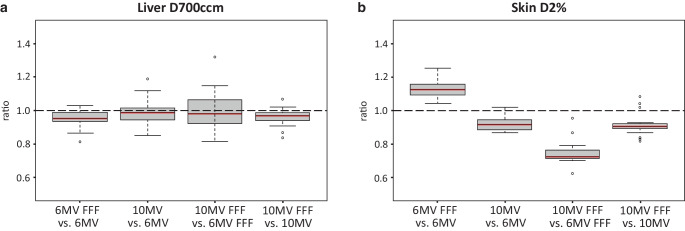
Organs at risk (OAR) of the liver lesions. Pairwise relative comparison for all four techniques: **a** liver D700ccm, **b** skin D2%

In terms of OAR, we found a dose saving for D700ccm of the liver for both FFF beams relative to the same energies with FF (Fig. 6a). In addition to lower dose exposures to the myelon as well as to the left kidney for 6 MV-FFF compared to 6 MV, we achieved dose savings to the ribs and sternum at 10 MV-FFF relative to 6 MV-FFF. Skin dose (D2%) also showed differences like the peripherally localized pulmonary lesions among the four treatment techniques applied (Fig. 6b).

## Discussion

In recent years, FFF beams have been increasingly implemented into clinical practice and have shown decisive advantages over flattened beams, especially in SBRT. As a result, several treatment planning studies already investigated the dosimetric effects of FFF beams for SBRT under VMAT conditions in clinical practice. In contrast to the majority of studies performed to date, we used energy-matched FFF beams and a wide range of target volume sizes, comparing four treatment techniques of 6‑ and 10-MV beams with and without FF under VMAT conditions.

Regarding the BOT, a mean time saving of 71.7% and 83.7% in the case of lung lesions and a mean time saving of 63% and 78.4% in the case of liver lesions were demonstrated for 6 MV-FFF and 10 MV-FFF, respectively, relative to the same energy with FF in each case. Thereby, the increasing risk of interplay effects with shortening BOT was countered by the use of multiple arcs and ≥ 2 fractions [37]. Thus, intrafractional movements of the patient or of the tumor can be minimized and techniques such as ABC can be performed in a more patient-friendly manner [13–15]. Our findings are consistent with previous studies demonstrating > 50% of BOT reduction for FFF beams, which is largely due to the higher dose rates that can be achieved with FFF beams [17, 22, 23, 26, 38]. Despite the nominally higher maximum dose rate of 10 MV-FFF, we observed only 8% and 11.3% shorter BOT compared to 6 MV-FFF for lung and liver lesions, respectively. Similar results were also obtained by Hrbacek et al. [23], who showed an average 13.3% shorter BOT for 10 MV-FFF compared to 6 MV-FFF using the Varian Truebeam linac, which has maximum dose rates of 1400 MU/min and 2400 MU/min for 6 MV-FFF and 10 MV-FFF, respectively.

When considering dose rate efficiency, a median of 95.4%, 92.8%, 99.9%, and 81.5% for the lung lesions and a median of 94.2%, 89.8%, 99.8%, and 81.5% for the liver lesions of the maximum possible dose rates of 6 MV, 6 MV-FFF, 10 MV, and 10 MV-FFF were achieved, respectively. This matches the findings by Hansen et al. [22], who also used a Versa HD in their planning study for SBRT lung treatments and achieved similar dose rate efficiencies for 6 MV (97.3%) and 6 MV-FFF (89.2%) with dual arc. Consequently, the full potential of the higher dose rates, particularly at 10 MV-FFF, could not be realized, as MLC and gantry speed constraints were limiting factors [25].

Total MUs in lung lesions were significantly lower for FFF beams than for flattened beams of the same energy level, mainly due to the large number of small-volume PTVs. We were able to show that FFF beams in lung lesions up to a PTV of 30.3 ccm required fewer MUs than flattened beams of the same energy. Total MUs in liver lesions were not significantly different for FFF beams than for flattened beams of the same energy level, due to the very heterogeneous size distribution of PTVs. We were able to show a cut-off value of 40.6 ccm comparing 6 MV-FFF versus 6 MV. Comparable results could be shown for 10 MV versus 10 MV-FFF depending on the PTV size.

Previous IMRT planning studies of EBRT of prostate, head and neck, brain, and lung tumors already observed lower MUs for FFF versus flattened beams [39, 40]. However, these results were mainly due to the fact that the linacs were not recalibrated with respect to MUs after FF removal, which is the case for our linac. Hansen et al. [22] also demonstrated a reduction in MUs for energy-matched FFF beams compared to flattened beams in lung treatments with a median PTV of 39.4 ccm (range: 10.8–87.5 ccm). In studies in which energy-matched FFF beams were not used, either no significant differences or significantly more MUs were observed for FFF beams (non-energy-matched). Referring to two studies with a PTV mean of 13.3 ccm (maximum size: 41.1 ccm) and a PTV median of 13.5 ccm (maximum size: 31.3 ccm) investigating mainly small peripheral lung lesions, no significant differences in total MUs between FFF and flattened beams could be demonstrated [28, 31]. Planning studies with a PTV median of 34 ccm (maximum size: 144.5 ccm) and 59.5 ccm (maximum size: 372.43 ccm) examining NSCLCs observed significantly more MUs with FFF versus flattened beams [23, 30]. Another study evaluating liver lesions with a PTV median of 164 ccm (maximum size: 435.1 ccm) showed significantly more MUs with FFF beams compared to flattened beams as well [38].

Liu et al. [30], using the smallest PTV size of 19.01 ccm, did not find a significant correlation between MUs and PTV. Indeed, Vieillevigne et al. [29] as well as Hrbacek et al. [23] showed a systematic increase in MU ratio (FFF/FF) with increasing PTV size.

Considering the graphical presentation of the output factor as a function of field size, as shown by Paynter et al. [32], a comparable phenomenon can be observed. At small field sizes, flattened beams require more MUs per Gy. The beam profile of flattened and FFF beams hardly differs at small field sizes, and FFF beams exhibit lower scattering. As the field size increases, flattened beams require fewer MUs per Gy compared to FFF beams. With increasing field size, the different beam profiles between flattened and FFF beams also become relevant, such that for FFF beams more MUs are needed to achieve the same dose coverage due to the conical beam profile. Apart from this effect being more pronounced in energy-matched than for non-energy-matched beams [32], other vendor- (e.g., MLC design, TPS, segmentation algorithm [41]) and patient-specific differences (e.g., PTV size/geometry, PTV distance from OAR) should also be noted.

Comparable target volume coverage was achieved for all plans. Nevertheless, for the lung lesions we showed a significant, but low improvement in GTV coverage for FFF beams and a significant better PTV coverage for 10 MV-FFF compared to 10 MV. For liver lesions, we found slightly superior GTV coverage for 10 MV-FFF compared to 10 MV and 6 MV-FFF, and better PTV coverage for FFF beams.

This is in line with the findings of Tambe et al. [24] and Liu et al. [30], who also demonstrated a minimally superior PTV coverage (< 1%) for FFF beams. By contrast, other studies could not detect any differences between flattened and FFF beams with respect to target volume coverage [22, 23, 26].

With regard to PTV conformity, no significant differences were found between FFF and flattened beams in lung and liver lesions, which is consistent with other results [22, 26, 29, 31, 38], whereas Hrbacek et al. [23] demonstrated significantly better conformity for FFF beams in their investigations. It should be noted that different CIs were used in each study, making the results comparable only to a limited extent.

Due to the very small target volumes in our patients with lung lesions, it was difficult to achieve CIs smaller than 1.2, which Vieillevigne et al. [29] also described. For the larger liver lesions, most CI values were < 1.2.

With respect to dose fall-off, our data suggest that the largest differences occur in the medium to lower dose range. Apart from the 6 MV-FFF GI_P&L_ being preferable over that for 10 MV-FFF in lung lesions, no significant differences for the GIs between the beam selections were found in lung and liver lesions. Barbiero et al. ([31]; GI = PIV_50%PIV_/PIV_95%PIV_) and Zhang et al. ([25]; GI = GI_P&L_) also came to the same conclusion when evaluating their GIs. By contrast, other studies showed significantly lower GIs (GI_RTOG_ [22–24, 28], GI_P&L_ [29]) for FFF versus flattened beams.

Our results for lung lesions indicated a significantly steeper dose fall-off from the prescribed dose to shell 2 and 3 for FFF beams. In addition, the integral dose (D50% and Dmean) was significantly lower for FFF versus flattened beams of the same energy in lungs as well as liver lesions, which matches the observations made by Hansen et al. [22].

These findings can be explained by the reduction of scattering, leaf transmission, and head leakage, which lead to lower out-of-field dose for FFF compared to flattened beams [18–20]. Furthermore, when comparing 6 MV-FFF and 10 MV-FFF, we found a lower dose exposure of healthy tissue for 6 MV-FFF.

The evaluation of the OAR of the lung cohort was performed in two subgroups that, to our knowledge, no study has investigated before. In the first subgroup, with centrally or superiorly located lung lesions, a tendency of a lower dose exposure to the contralateral lung for 6 MV-FFF as well as significant results for 10 MV-FFF relative to the irradiation technique of the same energy level with FF were found. This was also the case in the second subgroup, the peripheral lung lesions, for 6 MV-FFF versus 6 MV.

Hansen et al. [22] and Tambe et al. [24] reported comparable results, but also showed significant dose savings for 6 MV-FFF versus 6 MV in the tumorous lung, which cannot be confirmed by our data so far. In addition, for the first subgroup 10 MV-FFF offered superior dose savings of the total lung and myelon compared to 10 MV. Consistent with the results of Lu et al. [27] and Tambe et al. [24], we found a significantly higher dose exposure to the lung for 10 MV-FFF versus 6 MV-FFF. In peripheral lung lesions, comparable results were found with respect to the Dmean of the total as well as tumorous lung.

Regarding liver lesions, a significantly lower dose exposure to the liver (D700ccm) was demonstrated for FFF beams compared to flattened beams of the same energy level. This is consistent with the results of Vieillevigne et al. [29]. Further dose savings in OAR (i.e., myelon, kidney left) of liver lesions were achieved for 6 MV-FFF compared to 6 MV. Overall, significant differences were observed especially in OAR in the lower dose range, as was already shown by our findings regarding dose fall-off in healthy tissue. Nonetheless, even a small dose reduction is worthwhile and lowers the risk of secondary cancers [42].

Compared to the other beam selections, 10 MV-FFF showed the lowest dose exposure of the skin. With 6 MV-FFF, on the other hand, we found the highest average skin dose. Other studies also indicate superior skin protection for 10 MV-FFF compared to 6 MV-FFF and 6 MV [15, 24, 43].

Due to the lack of beam hardening effect and the resulting softer photon spectrum with FFF beams, a higher skin dose can be expected. This is partially offset by the reduction in head scatter and leaf leakage due to the absence of the FF, which contributes to a lower surface dose [44].

Since energy-matched FFF beams were used in our study and consequently have a higher energy than unmatched FFF beams, we found a skin dose saving even for 10 MV-FFF versus 10 MV. On the other hand, for 6 MV-FFF versus 6 MV, the skin dose was mostly higher. Apparently, for energy-matched 6 MV-FFF the missing beam hardening effect still outweighs the favorable characteristics such as reduction in head scatter, head leakage, and leaf transmission of the FFF beams.

### Study limitations

Limitations of our study are mainly due to the retrospective design and the limited patient population included in our liver cohort. Nevertheless, our investigations show the influence of vendor-specific (e.g., linac, MLC, gantry speed, TPS, dose calculation algorithm) as well as patient-specific (e.g., PTV size/geometry, distance to OAR, irradiation scheme) differences in the use of FFF beams in SBRT. This is of great interest for personalized medicine.

In this context, the multicenter planning studies of the DGMP Working Group have shown that these dosimetric differences depend more on techniques (IMAT, 3D-CRT, SF-IMRT, robotic radiosurgery) than on the planners [45]. The multiparameter dose prescription with normalization to GTV or ITV mean or median dose, pointed out by the authors, can harmonize the differences between different energies, flattened and unflattened, and matched and unmatched FFF beams, leading to further standardization of SBRT [45, 46].

## Conclusion

We found that flattening filter-free (FFF) beams show significant time savings in beam-on time (BOT) with equal to slightly superior target volume coverage and sparing of healthy tissue as well as organs at risk (OAR) in both lung and liver lesions, by comparing energy-matched flattened and FFF beams of two energies (6 MV and 10 MV) under volumetric modulated arc therapy (VMAT) conditions in a comprehensive stereotactic body radiotherapy (SBRT) planning study. Our findings suggest that for smaller planning target volumes (PTVs) fewer monitor units (MUs), whereas for larger PTVs a higher number of MUs, are usually required for FFF beams compared to flattened beams of the same energy level. With only slightly longer BOT, 6 MV-FFF showed superior dose savings over 10 MV-FFF, especially in the lung itself, making it our choice for SBRT of lung lesions. For liver lesions, we would prefer 10 MV-FFF over 6 MV-FFF because of the shorter BOT and no significant dose savings in the major OAR. Furthermore, this study highlights the dosimetric differences to be considered between energy-matched and non-energy-matched FFF beams.

### Supplementary Information

Additional tables, List of abbreviations
